# Molecular Mechanism of Phosphorylation-Mediated Impacts on the Conformation Dynamics of GTP-Bound KRAS Probed by GaMD Trajectory-Based Deep Learning

**DOI:** 10.3390/molecules29102317

**Published:** 2024-05-15

**Authors:** Jianzhong Chen, Jian Wang, Wanchun Yang, Lu Zhao, Juan Zhao, Guodong Hu

**Affiliations:** 1 School of Science, Shandong Jiaotong University, Jinan 250357, China; wangjian_lxy@sdjtu.edu.cn (J.W.); yangwch1982@126.com (W.Y.); zhaolusdu@163.com (L.Z.); zhjuan2002@126.com (J.Z.); 2 Shandong Key Laboratory of Biophysics, Institute of Biophysics, Dezhou University, Dezhou 253023, China

**Keywords:** KRAS, Gaussian accelerated dynamics simulations, deep learning, phosphorylation, free energy landscape

## Abstract

The phosphorylation of different sites produces a significant effect on the conformational dynamics of KRAS. Gaussian accelerated molecular dynamics (GaMD) simulations were combined with deep learning (DL) to explore the molecular mechanism of the phosphorylation-mediated effect on conformational dynamics of the GTP-bound KRAS. The DL finds that the switch domains are involved in obvious differences in conformation contacts and suggests that the switch domains play a key role in the function of KRAS. The analyses of free energy landscapes (FELs) reveal that the phosphorylation of pY32, pY64, and pY137 leads to more disordered states of the switch domains than the wild-type (WT) KRAS and induces conformational transformations between the closed and open states. The results from principal component analysis (PCA) indicate that principal motions PC1 and PC2 are responsible for the closed and open states of the phosphorylated KRAS. Interaction networks were analyzed and the results verify that the phosphorylation alters interactions of GTP and magnesium ion Mg^2+^ with the switch domains. It is concluded that the phosphorylation pY32, pY64, and pY137 tune the activity of KRAS through changing conformational dynamics and interactions of the switch domains. We anticipated that this work could provide theoretical aids for deeply understanding the function of KRAS.

## 1. Introduction

RAS proteins, a group of small GTPases, play vital roles in intracellular signal transduction pathways and are responsible for cell growth, proliferation, differentiation, and survival [1,2,3,4]. In fact, the RAS proteins primarily include four isoforms in human cells, namely HRAS, NRAS, KRAS4A, and KRAS4B. They function as a molecular switch by cycling between the GTP-bound active state and the GDP-bound inactive one [5,6,7,8]. Recently, increasing attention has been paid to these RAS proteins due to their close relations to a variety of human cancers. Previous studies have indicated that mutant RAS proteins with a constitutively active state trigger tumorigenesis by hyperactivating downstream signaling pathways. Among HRAS, NRAS, and KRAS, KRAS is involved in the most frequently mutated oncogene in human cancers, accounting for approximately 25% of lung, 40% of colorectal, and 95% of pancreatic cancers [9]. Overall, KRAS oncogenes make up about 82% of the Ras nonsynonymous mutations found in human oncogenes [10]. Therefore, KRAS has been given special attention and is of interest in understanding treatment strategies for human cancers.

**Figure 1:**
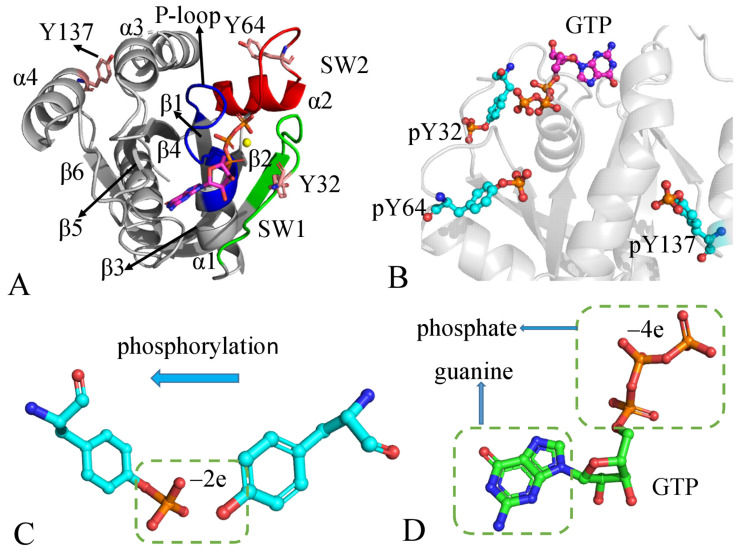
Molecular structure: (**A**) the GTP-bound WT KRAS, in which secondary structure and phosphorylation group are labeled, (**B**) the geometry position of the phosphorylated residues relative to GTP, (**C**) phosphorylation with two net negative charges, and (**D**) GTP. In this Figure, KRAS is shown in cartoon modes while GTP and phosphorylation-related residues are shown in ball-stick modes.

KRAS has similar structures to HRAS and NRAS, primarily composed of the effector lobe and allosteric lobe. The effector lobe, including residues 1–86, is located at the N-terminal region of the catalytic domain, which forms three common secondary structures, namely the P-loop (9–18), switch domain 1 (SW1: residue 28–40), and switch domain 2 (SW2: residue 59–75), as depicted in Figure 1A. The allosteric lobe, including residues 87–166, consists of an α-helical dimerization interface and nucleotide base binding motifs (Figure 1A). In terms of function, the effector lobe participates in the binding of KRAS to GTPase activating proteins (GAPs) and guanosine exchange factors (GEFs), while the allosteric lobe is responsible for relaying information through protein interactions [11,12,13,14,15,16]. Point mutations and residue modifications can directly affect the activity of KRASB by disturbing the conformational dynamics of the switch domains SW1 and SW2 [17,18,19,20,21,22,23,24,25,26]. Furthermore, point mutations in codons 12, 13, or 61 convert RAS into an active oncoprotein by impeding the GTPase activity of RAS and changing the conformational states of the switch domains [9,27,28,29,30,31,32,33,34,35,36]. Based on the roles of conformational dynamics for SW1 and SW2, clarifying the molecular mechanism of the alterations in conformational behavior of KRAS caused by mutations and residue modifications remains a significant and outstanding aim because of its involvement in a variety of cancers.

Phosphorylation, one of the residue modifications, is a common occurrence in proteins within specific environments, which can impact the functionality of proteins. Recent studies have focused on exploring the effects of phosphorylation on the activity of RAS proteins [37,38]. Kano et al. demonstrated that KRAS phosphorylation through Src on residues Y32 and Y64 changes the conformation of the SW1 and SW2, which stalls multiple steps of the GTPase cycle and disturbs binding of KRAS to effectors [39]. Wang et al. proposed that phosphorylation of Y32 and Y64 influences the catalytic transformations between GTP- and GDP-bound states, as mediated by the intrinsic or GAP/SOS (Son of sevenless) and affects its signal transduction to Raf [2]. Khaled et al. employed all-atom molecular dynamics simulations (MD) and Markov state models to conduct a comparative analysis of the oncogenic G12D KRAS mutant and its phosphorylated variant at Y32. Their results revealed that the G12D KRAS with Y32 phosphorylation exhibits greater flexibility in the SW1 compared to the G12D KRAS [40]. The work of Qiu et al. highlighted the significance of phosphorylation at multiple residues in regulating both wild-type (WT) and mutated RAS proteins, shedding light on the mechanistic insights into associated signaling pathways [41]. Although a deeper understanding of RAS phosphorylation can provide valuable insights for future therapeutic strategies, the current knowledge regarding the phosphorylation-mediated impacts on KRAS activity remains limited. Therefore, further investigation into the influence of phosphorylation on the conformational dynamics of KRAS is essential for the future design of anticancer therapies.

Among the current tools for probing the conformational dynamics of targets, conventional molecular dynamics (cMD) [42,43,44,45,46,47,48], Gaussian accelerated molecular dynamics (GaMD) simulations [49,50,51], and analysis of binding free energy [52,53,54,55,56,57] have been extensively used to reveal the molecular mechanisms and free energy profiles underlying conformational changes. Compared to cMD simulations, GaMD simulations more easily overcome energy barriers in protein systems. Furthermore, GaMD simulations have obtained successes in probing alterations in the conformational dynamics of targets [58,59,60,61,62,63,64]. Despite the great advantages of cMD and GaMD, there is a significant challenge in efficiently extracting dynamic information and functional differences of targets from a large MD trajectory. To address this issue, cMD and machine learning (ML) were integrated to deeply decode the function of G protein-coupled receptors (GPCRs) from the MD trajectory [65,66]. Miao’s group proposed a trajectory-based deep learning (DL) approach, called GaMD, DL, and free energy profiling workflow (GLOW), to successfully decipher the molecular mechanisms underlying the activation and allosteric modulation of G protein-coupled receptors [67,68]. The aforementioned methods provide reliable tools for gaining insights into the phosphorylation-mediated effects on the conformational dynamics of KRAS.

To achieve our goal, we selected the GTP-bound WT KRAS and the GTP-bound phosphorylated KRAS at residues Y32, Y64, and Y137 to investigate the influence of phosphorylation on KRAS conformations. The phosphorylation sites and sidechain changes are shown in Figure 1B,C, along with the structures of GTP displayed in Figure 1D. It is evident that a phosphate group on the sidechain of Tyr can have a greater effect due to its size and negative charge [37]. Phosphorylation of Y32 and Y64 significantly affects the binding of KRAS to its effectors. Phosphorylation of Y137 induces allosteric changes in protein conformation and effector binding [2,39], providing a mechanism for effector-initiated modulation of RAS signaling. However, the molecular mechanism underlying the effects of phosphorylation currently remains unclear, which is why we selected these four systems. In this work, multiple independent GaMD (MI-GaMD) simulations were conducted to enhance the conformational sampling of KRAS and DL was employed to identify the phosphorylation-sensitive sites. Additionally, principal component analysis (PCA) [69,70,71] and calculations of free energy landscapes (FELs) were performed to reveal changes in conformational dynamics and free energy profiles induced by phosphorylation at these three positions. We anticipate that this study will provide valuable insights into the regulation of KRAS activity.

## 2. Results and Discussion

### 2.1. Phosphorylation-Mediated Difference in Domain Contacts Revealed by Deep Learning

**Figure 2:**
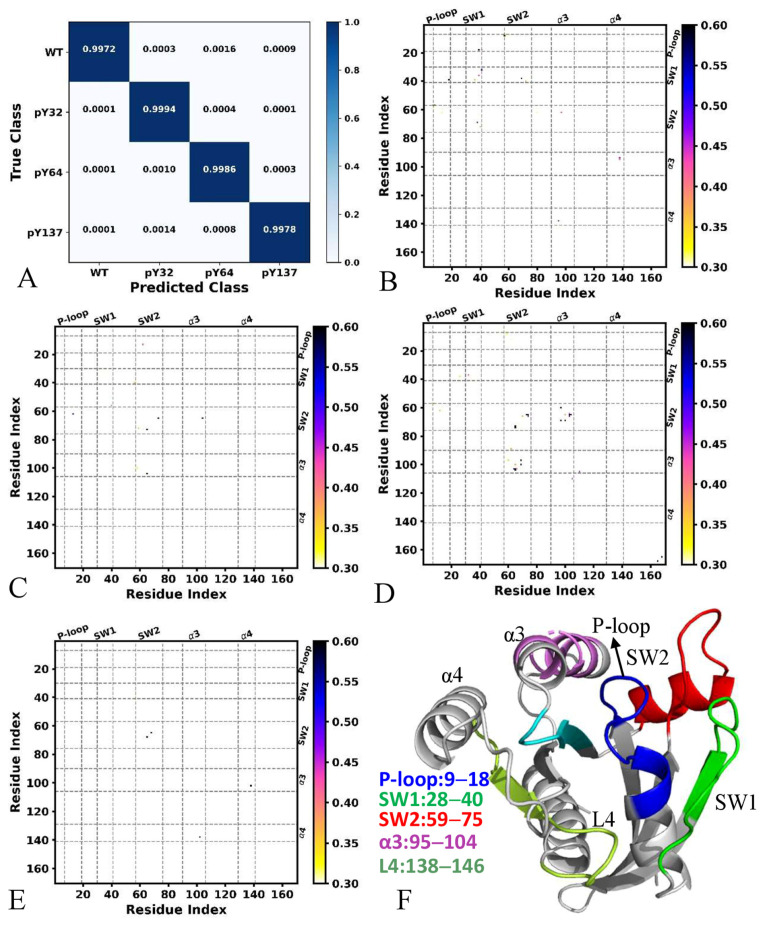
Classification and saliency map of residue contact gradients: (**A**) classification of the GTP-bound WT, pY32, pY64, and pY137 KRAS, (**B**–**E**) the saliency map of residue contact gradients for the GTP-bound WT, pY32, pY64, and pY137 KRAS, and (**F**) key structural domains revealed by the DL. The gradient of each residue contact is shown in a 0.3 (white) to 0.6 (black) color scale.

MD trajectory-based DL has been used to explore molecular mechanisms underlying the activation and allosteric modulation of G protein-coupled receptors [67,68]. Residue contacts were calculated to produce images for the DL. Classification of the GTP-bound WT, pY32, pY64, and pY137 KRAS were achieved by the DL and the results were shown in Figure 2. The overall accuracy and loss reached on the validation set after 25 epochs are 0.9982 and 0.0046, respectively (Supporting Information Figure S1). This high accuracy should be due to the high efficiency of conformational sampling performed by GaMD simulations. From the 8000 snapshots for the validation set of each system, the DL accurately classified most of four current systems (Figure 2A), including 7984 snapshots of the GTP-WT KRAS, 7988 snapshots of the GTP-pY32 KRAS, 7972 snapshots of the GTP-pY64 KRAS, and 7991 snapshots of the GTP-pY137 KRAS individually. As shown in Figure 2A, only a marginal portion of the snapshots were inaccurately classified. In detail, ~0.01% (1 snapshots), ~0.04% (3 snapshots), and ~0.15% (12 snapshots) of the GTP-WT KRAS were, respectively, predicted as the GTP-pY32, pY64, and pY137 KRAS. The ~0.03% (3 snapshots), 0.04% (3 snapshots), and 0.08% (6 snapshots) of the GTP-pY32 KRAS were inaccurately classified as the GTP-WT, pY64, and pY137 KRAS, respectively. The ~0.16% (13 frames), ~0.05% (4 frames), and ~0.13% (11 frames) of the GTP-pY64 KRAS were inaccurately predicted as the GTP-WT, pY32, and pY137 KRAS, respectively, while the ~0.05% (4 frames), 0.03% (2 frames), and ~0.04% (3 frames) of the GTP-pY137 KRAS were erroneously recognized as the GTP-WT, pY32, and pY64 KRAS, separately. These results indicate that the information revealed by our current DL is reliable.

The pixel-attributed residue contact gradient maps of the most populated KRAS structures were depicted at Figure 2B,E. On the whole, the characteristic residue contacts of the GTP-WT KRAS are located between the SW1 and SW2, the P-loop and SW2, the helixes α3 and α4, as well as the helix α3 and SW2 (Figure 2B,F). The characteristic residue contact of the GTP-pY32 KRAS is primarily situated between the SW1 and the P-loop (Figure 2C,F). Compared to the GTP-WT KRAS, the phosphorylation pY32 leads to the disappearance of the characteristic residue contact between the SW1 and SW2 as well as α3 and α4 together with the α3 and SW2. The characteristic residue contacts of the GTP-pY64 KRAS were detected between the P-loop and SW1 as well as the P-loop and SW2 together with the α3 and SW2 (Figure 2D,F). By comparison with the GTP-WT KRAS, the phosphorylation pY64 results in the disappearance of the characteristic residue contact between the SW1 and SW2 as well as the α3 and α4. The characteristic residue contacts of the GTP-pY137 were located between the helixes α3 and α4 (Figure 2E,F). By referencing GTP-WT KRAS, the phosphorylation pY137 leads to the disappearance of the characteristic residue contacts between the SW2 and P-loop as well as the SW1 and SW2 together with the α3 and SW2.

Through the characteristic residue contacts learned by our DL, the phosphorylation-mediated impacts are involved in the structure domains SW1, SW2, α3, and α4 of KRAS. It is worth noting that the SW1 and SW2 participate in binding of KRAS to its effectors or regulators while the α3 and α4 are involved in allosteric regulations on the activity of KRAS [72]. The tyrosyl phosphorylation of KRAS disturbs the GTPase cycle through conformational alteration of the SW1 and SW2 [39], which agrees with our findings learned by the DL. The phosphorylation pY32 highly affects the flexibility of the SW1 and SW2 conformation and tunes the activity of KRAS [40]. The RAS phosphorylation at Y137 allosterically changes protein conformation and effector binding, revealing a mechanism for effector-initiated modulation of the RAS signaling [37]. These previous works support our current findings well.

### 2.2. Free Energy Profiles Affected by Phosphorylation

**Figure 3:**
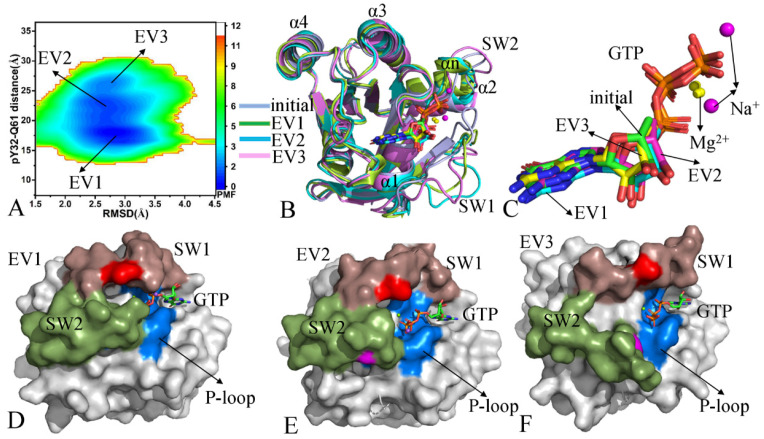
Free energy profiles and representative structures of the GTP-pY32 KRAS: (**A**) FEL, (**B**) superimposition of initial optimized structure with the EV1, EV2, and EV3 structures, (**C**) structural superimposition of GTP and magnesium ions (Mg^2+^) in the initially optimized structure and the EV1, EV2, and EV3 structures, and (**D**–**F**) geometric positions of the P-loop, SW1, and SW2 in the EV1, EV2, and EV3 structures, in which KRAS was shown in surface modes. The PMF is scaled in kcal/mol.

**Figure 4:**
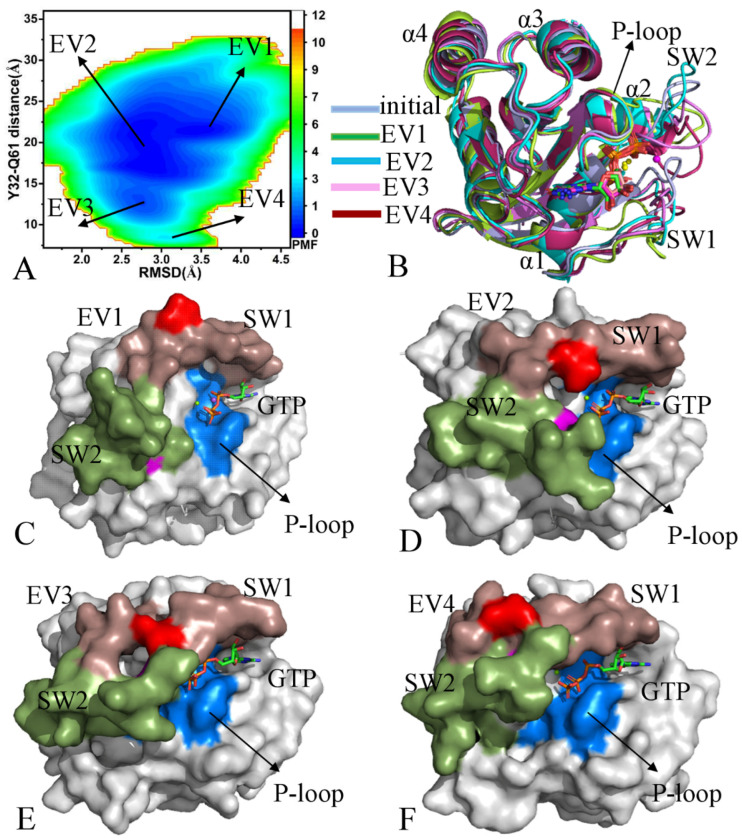
Free energy profiles and representative structures of the GTP-pY64 KRAS: (**A**) FEL, (**B**) superimposition of initial optimized structure with the EV1, EV2, EV3, and EV4 structures, and (**C**–**F**) geometric positions of the P-loop, SW1, and SW2 in the EV1, EV2, EV3, and EV4 structures, in which KRAS was shown in surface modes. The PMF is scaled in kcal/mol.

**Figure 5:**
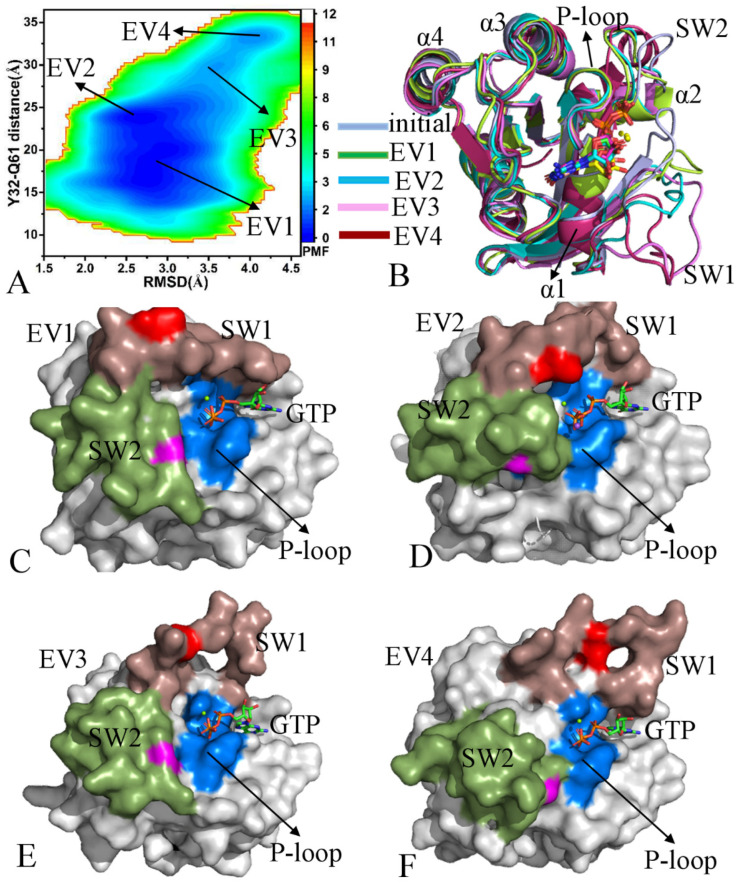
Free energy profiles and representative structures of the GTP-pY137 KRAS: (**A**) FEL, (**B**) alignment of initial optimized structure with the EV1, EV2, EV3, and EV4 structures, and (**C**–**F**) geometric positions of the P-loop, SW1, and SW2 in the EV1, EV2, EV3, and EV4 structures, in which KRAS was shown in surface modes. The PMF is scaled in kcal/mol.

The previous DL suggests that the residue contacts between the switch domains are heavily affected by the phosphorylation of three residues: Y32, Y64, and Y137. The distance of the Cα atom of residue 32 in the SW1 away from that of Q61 in the SW2 was selected as an RC. The root-mean-square deviations (RMSDs) of backbone atoms were calculated and the results were provided in Figure S2. It was noted that RMSDs can reflect the total structural fluctuation of KRAS, which is selected as another RC. Thus, these two RCs were used to build FELs to reveal the energy basis of the phosphorylation-mediated impacts on the conformations of KRAS. The FELs and the corresponding representative structures are depicted in Figure 3, Figure 4, Figure 5 and Figure S3–S6.

For the GTP-bound WT KRAS, only an energy valley (EV1) was captured by GaMD simulation and it is situated at the (RMSD, distance) of (2.8, 19.2) Å (Figure S3A), implying that GaMD simulations do not lead to conformational rearrangement of KRAS. To compare the structural difference between the crystal structure and the low energy structure EV1, the initial optimized structure was superimposed with the structure EV1 (Figure S3B). It is observed that only the SW1 produces a small deviation while GTP and magnesium ion (Mg^2+^) are aligned well (Figure S3C). The distance between Y32 and Q61 in the structure EV1 is only greater than 0.8 Å compared to that in the initial optimized structure (Figure S3D,E). These results suggest that the GTP-bound WT KRAS maintains structural stability through the entire GaMD simulation.

With respect to the GTP-bound pY32 KRAS, three energy valleys (EV1–EV3) were identified by GaMD simulations (Figure 3A). The EV1, EV2, and EV3 are, respectively, situated at the (RMSD, distance) of (2.8, 18.1), (2.6, 22.7), and (2.6, 26.1) Å (Figure 3A and Figure S4). The superimposition of the initially optimized structure with the representative structures EV1–EV3 shows that the SW1 evidently deviates from each other among different structures and has highly disordered states while the SW2 produces a small deviation (Figure 3B). On the contrary, GTP and magnesium ions (Mg^2+^) are aligned well among different structures (Figure 3C), indicating that they are stable during GaMD simulations. Compared to the GTP-bound WT KRAS, pY32 leads to big structural deviations of SW1 relative to the initially optimized structure and induces more disordered conformational states of the SW1 (Figure 3B). The switch domains of the EV1 structure form a closed state (Figure 3D), those of the EV2 structure show a semi-open one (Figure 3E), and those of the EV3 structure have an open state (Figure 3F). Based on these results, the pY32 results in the conformational transition of the switch domains between the closed and open states. It is well known that SW1 is involved in the binding of KRAS to its effectors. Therefore, pY32 affects the KRAS-effector and GTP-KRAS binding, which regulates the activity of KRAS. In addition, two sodium ions (Na^+^) appear near the phosphate group of GTP (Figure 3C).

With regard to the GTP-bound pY64 KRAS, four energy valleys EV1–EV4 were captured by GaMD simulations and they are located at the (RMSD, distance) of (3.4, 21.5), (2.7, 19.1), (2.7, 13.2), and (3.1, 11.8) Å, respectively (Figure 4A). The alignment of the initially optimized structure with the structures EV1-EV4 verifies that the switch domains SW1 and SW2 not only produce evident deviations but also possess highly disordered states (Figure 4B). By comparison, the disorder extent of SW2 is higher than that of SW1 (Figure 4B). However, GTP and magnesium ions (Mg^2+^) agree well with each other among the initially optimized structures and four representative structures (Figure S5A), reflecting that these two ligands are stably maintained in the binding pocket. The switch domains of the structure EV1 shows an open conformation (Figure 4C) while those of the structures EV2–EV4 have a closed conformation (Figure 4D,F); in particular, the structure EV4 verifies that pY64 leads to the conformational transition between the closed and open states of the switch domains. Among four representative structures, the switch domains of the EV4 are the most compact (Figure S5). Compared to the GTP-bound WT KRAS, pY64 induces more energy states and more disordered switch domains, which exerts significant influences on the KRAS-effector binding and the activity of KRAS.

In the case of the GTP-bound pY137 KRAS, four energy valleys EV1–EV4 were detected by GaMD simulations and they are individually trapped at the (RMSD, distance) of (2.8, 18.9), (2.6, 23.7), (3.5, 29.6), and (4.0, 33.1) Å (Figure 5A and Figure S6B–E). The alignment of four representative structures EV1-EV4 with the initially optimized structure demonstrates that the switch domains SW1 and SW2 obviously deviate from each other and are located in a highly disordered state; moreover, the disorder extent of the SW1 is much higher than that of SW2 (Figure 5B). Different from the switch domains, GTP and magnesium ions (Mg^2+^) are aligned well among the structures EV1–EV4 and the initial structure (Figure S6A), implying that pY137 hardly affects the stability of GTP and Mg^2+^ through the entire GaMD simulations. The switch domains of the structure EV1 show a slightly closed state (Figure 5C) while those of the structures EV2-EV4 are located in an open state (Figure 5D,F). In particular, the switch domains of the structure EV4 are completely separated and do not form any overlap (Figure 5F). Thus, pY137 induces the conformational transition between the closed and open states of the switch domains. By comparison with the GTP-bound WT KRAS, pY137 not only induces more energy states but also leads to more disordered states of the switch domains. Thus, pY137 certainly impacts the binding of KRAS to its effector and highly disturbs the activity of KRAS.

Based on our current results, the phosphorylation of pY32, pY64, and pY137 yield significant influences on conformations and functions of KRAS: (1) the phosphorylation induces more energy states and changes free energy profile of KRAS, (2) the phosphorylation leads to more disordered states of the switch domains and induces conformational transition between the closed and open states of the switch domains, and (3) the phosphorylation hardly affects the structural stability of GTP and magnesium ions during GaMD simulation. GaMD simulations capture the conformational transition between the open and closed states of the switch domains, which shows the high efficiency of GaMD conformation sampling. The switch domains participate binding of KRAS to its effectors or regulators. Therefore, the highly disordered states of the switch domains caused by the phosphorylation necessarily generate significant impacts on the activity of KRAS. Differently, pY32 and pY137 regulate the activity of KRAS by primarily changing the conformation of the SW1 while pY64 tunes the activity by mainly altering the conformation of the SW2. The work of Kano et al. verified that KRAS phosphorylation via Src on Y32 and Y64 alters the conformation of the SW1 and SW2 and impairs binding to effectors, supporting our current findings [39]. The study by Bunda et al. indicated that the phosphorylation of pY32 alters the positioning or conformation of the SW1 so that it no longer accommodates Raf binding [38], agreeing with our current results.

### 2.3. Dynamics Behavior of KRAS Influenced by Phosphorylation

**Figure 6:**
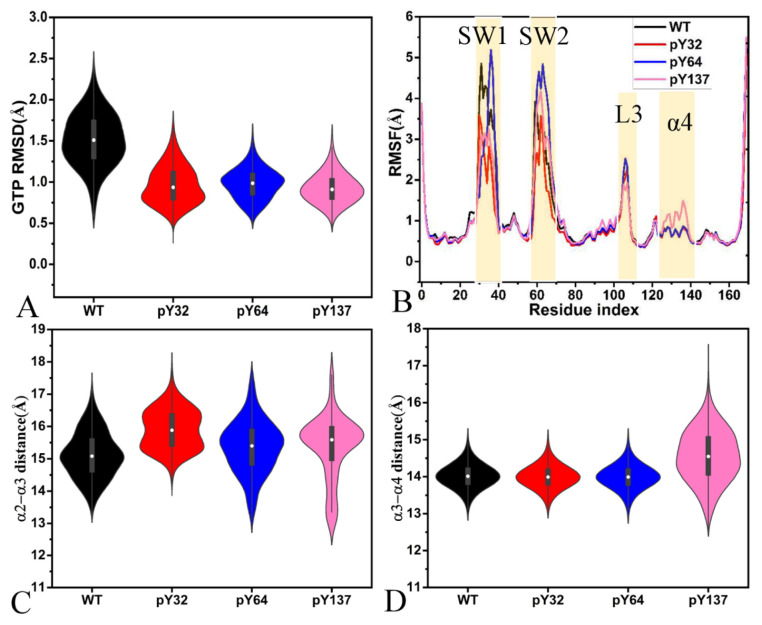
Dynamics indexes: (**A**) the probability distribution of GTP RMSDs, (**B**) RMSFs of KRAS, (**C**) the probability distribution of the distances between the mass center of all Cα atoms in helix α2 away from that of helix α3, and (**D**) the probability distribution of the distances between the mass center of all Cα atoms in helix α3 away from that of helix α4.

To understand the influences of the phosphorylation on structural stability of GTP, the RMSDs of heavy atoms for GTP were calculated relative to the initial structure (Figure S7). It is observed that GTP shows a stable fluctuation in four current systems. The RMSDs of GTP in the WT, pY32, pY64, and pY137 KRAS are populated at 1.5, 0.8, 1.0, and 0.9 Å (Figure 6A), respectively, indicating that pY32, pY64, and pY137 increase the structural stability of GTP in the binding pocket of KRAS. Root-mean-square fluctuations (RMSFs) of KRAS were estimated by using the coordinates of the Cα atoms (Figure 6B). The phosphorylation of pY32 and pY137 weakens the structural flexibility of the SW1 relative to the WT KRAS while pY64 slightly increases that of the SW1. The phosphorylation of pY64 and pY137 strengthens the structural flexibility of the SW2 compared to the WT KRAS but pY32 slightly reduces that of the SW2 (Figure 6B). By comparison with the WT KRAS, the phosphorylation pY64 increases the structural flexibility of the loop L3 linking the α3 and β5 while pY137 weakens that of this loop (Figure 1A and Figure 6B). Different from pY32 and pY64, pY137 obviously enhances the structural flexibility of the α4 (Figure 1A and Figure 6B). In fact, a common mechanistic basis inherent in the high flexibility of the switch regions relates to the conformational transition between two states, which has been revealed by the previous study [2,73].

To probe the effect of the phosphorylation on allosteric regulation, the distances between the mass centers of all Cα atoms in the helix α2 and α3 were computed (Figure S8A) and the results show a stable fluctuation. The distances between α2 and α3 are primarily distributed at 14.9, 15.9, 15.7, and 15.7 Å in the GTP-bound WT, pY32, pY64, and pY137 KRAS, respectively, (Figure 6C), verifying that pY32, pY64, and pY137 increase the distance between α2 and α3. In addition, the distances between the mass centers of all Cα atoms in the helix α3 and α4 were also calculated (Figure S8B) and the results indicate that this distance fluctuates at a range of 11.8–19.8 Å during GaMD simulations of four systems. The distances between α3 and α4 are distributed at 13.9 Å in the GTP-bound WT, pY32, and pY64 KRAS while this distance is populated at 14.5 Å with a wider distribution range in the GTP-bound pY137 KRAS (Figure 6D), suggesting that pY137 increases the distance between α3 and α4. Two helixes α3 and α4 form an interface of allosteric regulation; thus, the changes in distances of the α3 away from α2 and α4 induced by the phosphorylation affect the allosteric activity of KRAS.

**Figure 7:**
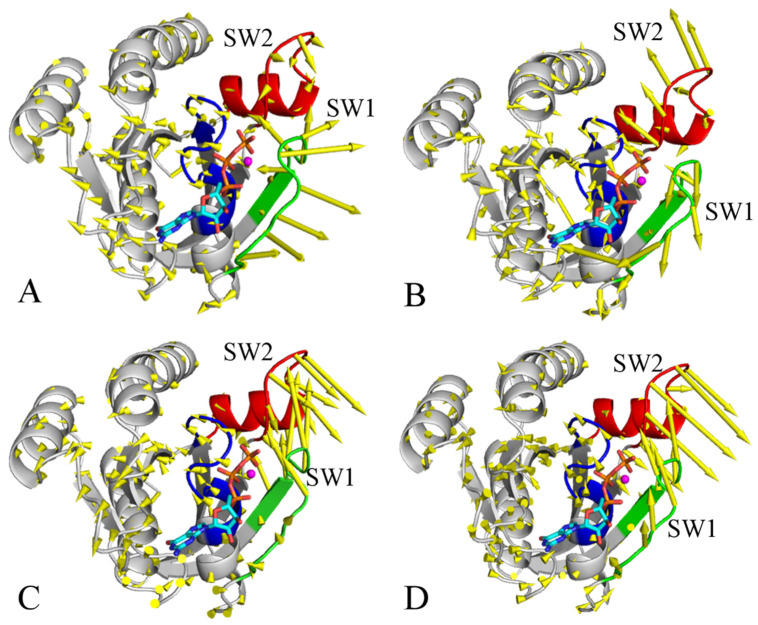
Concerted motions of the GTP-KRAS revealed by the PC1 from the PCA: (**A**) the GTP-WT KRAS, (**B**) the GTP-pY32 KRAS, (**C**) the GTP-pY64 KRAS, and (**D**) the GTP-pY137 KRAS. KRAS was shown in cartoon mode and GTP is shown in ball-stick modes. The blue, green and red indicate the P-loop, SW1 and SW2, respectively.

**Figure 8:**
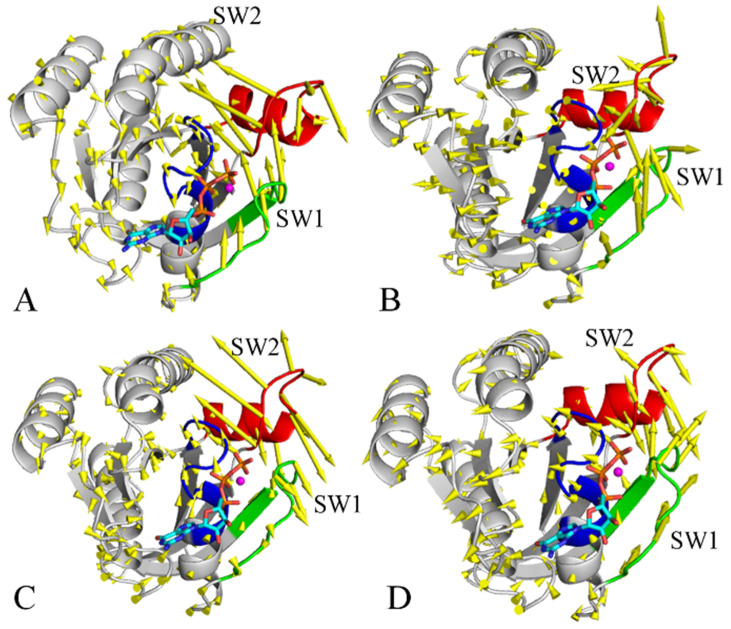
Collective movement of the GTP-KRAS revealed by the PC2 from the PCA: (**A**) the GTP-WT KRAS, (**B**) the GTP-pY32 KRAS, (**C**) the GTP-pY64 KRAS, and (**D**) the GTP-pY137 KRAS. KRAS was shown in cartoon mode and GTP is shown in ball-stick modes. The blue, green and red indicate the P-loop, SW1 and SW2, respectively.

To explore phosphorylation-mediated impacts on the dynamics behavior of KRAS, PCA was performed on the coordinates of the Cα atoms recorded at the SGT. The function of eigenvalues over eigenvectors is depicted in Figure S9, which is used to characterize structural fluctuation along the eigenvectors. The first eigenvalues of the pY32, pY64, and pY137 KRAS are obviously increased relative to the WT KRAS, hence the phosphorylation of pY32, pY64, and pY137 enhances the structural fluctuation of KRAS along the first eigenvector. It is also observed that the first two eigenvalues of the four systems are much higher than the other eigenvalues (Figure S8). To check the roles of the first two PCs (PC1 and PC2) in conformational dynamics, they were visualized by means of the VMD1.9.3 software [74] and the results corresponding to PC1 and PC2 were separately depicted in Figure 7 and Figure 8. The switch domains display well-concerted motions in the first two PCs; furthermore, the phosphorylation greatly affects the dynamics behavior of the switch domains. In the PC1 of the GTP-bound WT KRAS, the SW1 and SW2 have an opposite motion tendency and tend to be close to each other (Figure 7A). In the PC2 of the GTP-bound WT KRAS, the SW1 also has a motion tendency to be close to the SW2 (Figure 8A). This information agrees with the previous results that no conformation transition occurs between the closed and open states of the switch domains in the GTP-WT KRAS. In the PC1 of the GTP-pY32 KRAS, the switch domains have a tendency to open (Figure 7B) but it yields a closed tendency in the PC2 (Figure 8B). Therefore, in the GTP-pY32 KRAS, the PC1 is responsible for the open state of the switch domains while the PC2 mediates the closed state. Different from the GTP-pY32 KRAS, the switch domains of the GTP-pY64 and pY137 KRAS tend to be closed in the PC1 (Figure 7C,D) but they have an opening tendency in the PC2 (Figure 8C,D). It is concluded that the PC1 drives the closed conformation of the switch domains in the GTP-pY64 and pY137 KRAS while the PC2 is responsible for the open conformation of the switch domains. These results are in good agreement with the phosphorylation-mediated conformation transition between the open and closed states of the switch domains revealed by the previous analysis of FELs.

In summary, pY32, pY64, and pY137 highly influence the dynamics behavior and activity of KRAS: (1) the phosphorylation stabilizes the structure of GTP in the binding pocket of KRAS and affects the GTP-KRAS interactions, (2) the phosphorylation changes the relative geometric position of the helix α3 to α2 and α4, which affects the allosteric regulation of KRAS, and (3) the phosphorylation alters concerted motions of the switch domains and two principal components PC1 and PC2 are responsible for conformational transition between the open and closed states of the switch domains, which disturbs binding of KRAS to effectors or regulators. Our current findings are in basic agreement with the previous studies [2,40,75].

### 2.4. Dihedral Angle of Phosphorylated Residues

**Figure 9:**
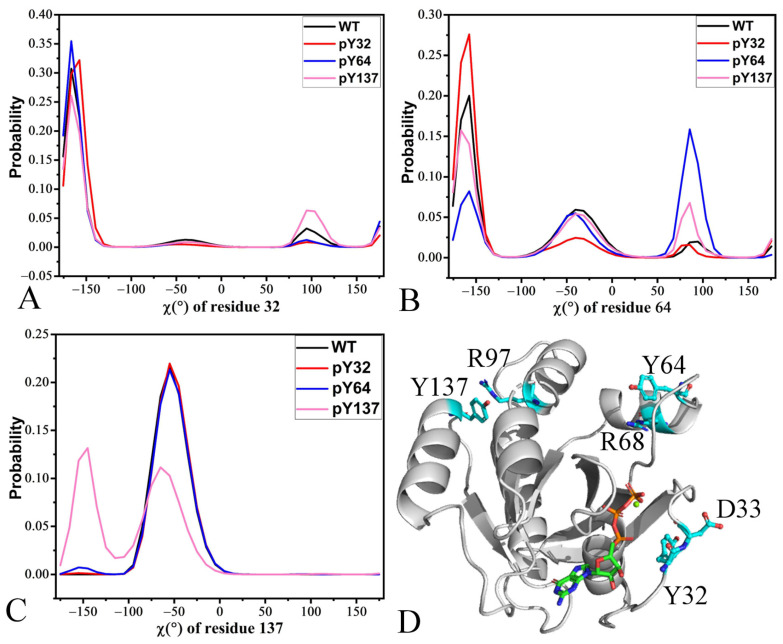
The probability of the χ angle for the sidechains of three phosphorylation-related residues: (**A**) residue 32, (**B**) residue 64, (**C**) residue 137, and (**D**) geometric positions of key residues.

The phosphorylation not only enlarges the sidechain of the phosphorylated residues but also brings two net negative charges for the sidechain. To clarify the molecular mechanism underlying the phosphorylation-mediated effect, the atoms N, C, CB, and CG of three residues 32, 64, and 137 were utilized to calculate the dihedral angles through the CPPTRAJ program in Amber. The probability distributions of these dihedral angles were plotted in Figure 9A–C and key residues near the phosphorylation sites were depicted in Figure 9D. Indeed, the phosphorylation changes the rotation and distribution of the sidechains of the phosphorylated residues.

The sidechain of residue 32 partly rotates from −166.9° to 96.1° to −157.1° due to pY32 and pY64 compared to the WT KRAS while that of residue 32 partly rotates from −166.9° to 94.7° because of pY137 (Figure 9A); thus, the phosphorylation changes the conformation distribution of the sidechain for residue 32. Structurally, Y32 is located near D33 in the WT KRAS (Figure 9D), hence the negative charges of pY32 yielding strong electrostatic repulsive interactions with the carbonyl group of D33. The negative charges of pY64 and pY137 also form long-range electrostatic repulsive interactions with the carbonyl group of D33. These electrostatic repulsive interactions drive the conformational arrangement of the sidechain for residue 32. The phosphorylation of Y32 leads to an electrostatic repulsion against the negatively charged D38 and D57 from the nucleotide-binding groove, which changes the SW1 positioning or conformation so that it no longer accommodates Raf binding [38].

The dihedral angles of the sidechain for residue 64 are mainly distributed at ~−157.4°, −39.5°, and 85.7° in four systems (Figure 9B). Compared to the WT KRAS, pY32 leads to a decrease in the distribution of the sidechain for residue 64 in −39.5° and 85.7° and an increase in the distribution in −157.4°. The phosphorylation of pY64 and pY137 leads to partial rotations from −157.4° to 85.7° by comparison with the WT KRAS (Figure 9B). Structurally, the sidechain of Y64 is next to the guanidino group of R68 with a net positive charge in the WT KRAS (Figure 9D). The negative charges of pY64 produce strong electrostatic attraction interactions with the guanidino group of R68 in the SW2, which drives the rotation of the sidechain of pY64 (Figure 9B). The negative charges of pY32 and pY137 also generate long-range electrostatic attraction interactions with the guanidino group of R68, which induces partial rotation of the sidechain for Y64. These electrostatic attraction interactions are responsible for the conformational arrangement of the sidechain for residue 64. The previous work indicated that the conformational arrangement of residue 64 caused by the phosphorylation possibly affects the association of RAS and RAN mediated by R68 and R76 with GEFs [76], which agrees with our current result.

The dihedral angles of the sidechain for Y137 are populated at −54.5° in the GTP-bound WT, pY32, and pY137 KRAS while the dihedral angle of pY137 is situated at two peaks, −145.7° and −64.5°, in the GTP-bound pY137 KRAS (Figure 9C). This result indicates that pY137 results in a rotation of the residue 137 sidechain from −64.5° to −147.5° compared to the WT KRAS. In the geometry position, Y137 in the helix α4 is located near R97 in the α3 in the WT KRAS. Thus, the negative charges of the sidechain in pY137 yield strong electrostatic attraction interactions with the guanidino group of R97 (Figure 9D), which makes the sidechain of pY137 rotate toward the gunnidio group of R97. This rotation of pY137 forms a steric hindrance effect with the α3, which increases the distance between the α3 and α4. The phosphorylation of Y137 triggers allosteric changes in GTP-bound RAS that enhance its affinity for RAF1-RBD and severely impair or even abolish the ability of RAS to hydrolyze GTP [37].

In summary, the phosphorylation of tyrosyl not only enlarges the sidechain of residue but also brings two net negative charges. This residue modification easily forms a steric hindrance effect with its neighboring secondary structure and also produces electrostatic attraction or repulsive interactions with the charged residues, which drives the conformational rearrangement of the phosphorylated residues. Thus, electrostatic interactions and steric hindrance effects caused by the phosphorylation of tyrosyl affect the binding of KRAS to effectors and allosteric changes in GTP-bound KRAS [37].

### 2.5. Interaction Networks Affected by Phosphorylation

**Figure 10:**
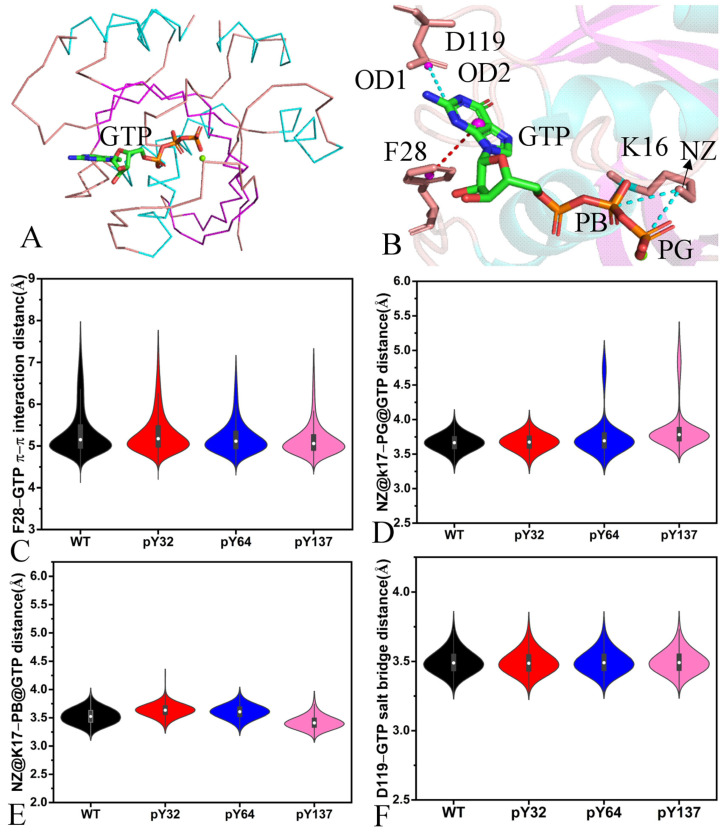
Key interactions of GTP with KRAS and their corresponding distance distributions. (**A**) Relative position of GTP in the binding pocket of KRAS, (**B**) geometry positions of key interactions, (**C**) the distances of the π-π interaction between GTP and F28, (**D**) the distances of the salt bridge interaction between the phosphorus atom PB of GTP and the nitrogen atom NZ of K16, (**E**) the distances of the salt bridge interaction between the phosphorus atom PG of GTP and the nitrogen atom NZ of K16, and (**F**) the distances of the salt bridge interaction between the guanine group of GTP and the carbonyl group of D119.

**Figure 11:**
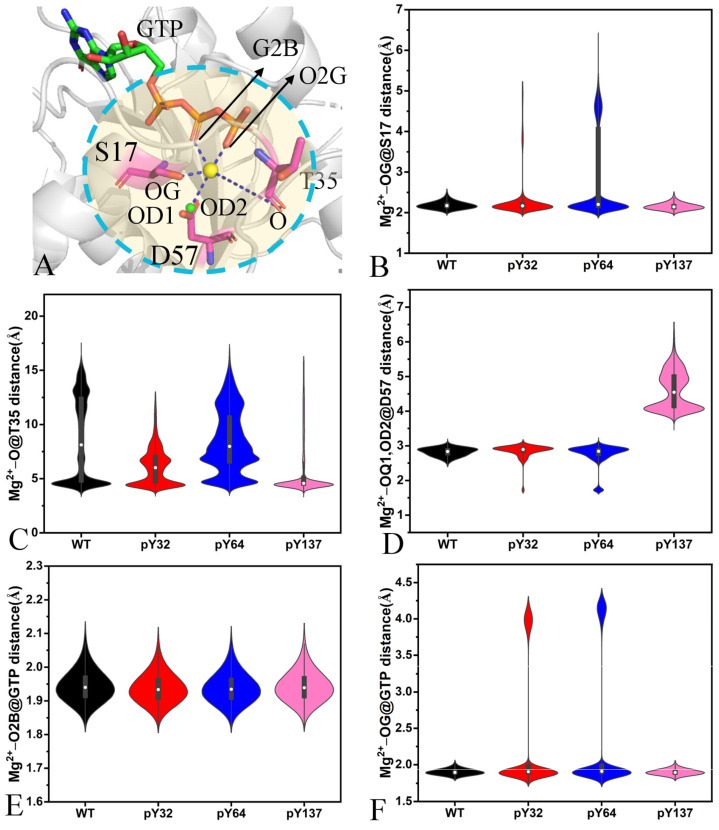
Key interactions of magnesium ions (Mg^2+^) with GTP and KRAS together with their distance distributions: (**A**) the geometry position of interactions, (**B**) the distances between Mg^2+^ and the oxygen atom OG of S17, (**C**) the distances of Mg^2+^ away from the oxygen atom O of T35, (**D**) the distances between Mg^2+^ and the oxygen atom O of T35, (**E**) the distances of Mg^2+^ away from the mass center of oxygen atoms of OD1 and OD2 in D57, (**F**) the distances of Mg^2+^ away from oxygen atom O2B of GTP, and (**F**) the distances between Mg^2+^ and oxygen atom OG of GTP.

To investigate the phosphorylation-mediated effect on the interaction networks of GTP with KRAS, the protein–ligand interaction profiler (PLIP) web server [77,78] was employed to identify GTP-KRAS interaction modes (Figure 10A,B and Figure S10). Moreover, the CPPTRAJ program was adopted to recognize the hydrogen bonding interactions (HBIs) of GTP with KRAS (Table 1). The distances involved in the salt bridge, π-π, and electrostatic interactions were computed and the corresponding results are illustrated in Figure 10B,E. Moreover, the distances of Mg^2+^-mediated electrostatic interactions with GTP and residues were also estimated and the relevant information was presented in Figure 11 and Figure S11.

According to Figure 10A, Figure 11B, and Figure S10, the guanine group of GTP forms a π-π interaction with the phenyl of F28. The distances between the mass center of the guanine group and that of phenyl are populated at 5.1 Å in four systems (Figure 11C), indicating that pY32, pY64, and pY137 hardly affect this π-π interaction. The phosphorus atom (PG) of GTP yields a salt bridge interaction with the nitrogen atom (NZ) of K16 (Figure 11B,C). The distances of PG away from NZ are located at 3.64 Å in the GTP-bound WT and pY32 KRAS (Figure 11D). This distance is distributed at 3.65 and 4.76 Å in the GTP-bound pY64 KRAS while it is populated at 3.54 and 4.68 Å in the GTP-bound pY137 (Figure 11D), showing that pY64 and pY137 weaken the stability of this salt bridge interaction. The phosphorus atom (PB) of GTP forms a salt bridge with the nitrogen atom (NZ) of K16 (Figure 11B). The distances between PB and NZ are distributed at 3.58, 3.64, 3.60, and 3.32 Å in the GTP-bound WT, pY32, pY64, and pY137 KRAS (Figure 11E), respectively, indicating that pY32 and pY64 hardly influence this salt bridge while pY137 slightly strengthens this salt bridge. The guanine group of GTP generates a salt bridge interaction with the carbonyl of D119 (Figure 11B) and the distances between the mass center of three nitrogen atoms (N1-N3) of GTP and that of oxygen atoms (OD1 and OD2) are populated at 3.49 Å (Figure 11F), verifying that the phosphorylation does not affect this salt bridge interaction.

Based on Table 1 and Figure S10, the phosphate group of GTP forms HBIs with six residues from the P-loop, including G13, V14, G15, K16, S17, and A18. Compared to the WT KRAS, pY64 obviously decreases the occupancy of hydrogen bonds between GTP and S17 while pY137 evidently reduces the occupancy of HBIs of GTP with V14 and S17 (Table 1). GTP produces HBIs with residues V29 and D30 from the SW1 and their occupancy is lower than 30.6% (Table 1), suggesting that these hydrogen bonds are highly unstable through the GaMD simulation. By comparison with the WT KRAS, the phosphorylation decreases the occupancy of HBIs of GTP with V29 and D30 in the SW1, which explains the reason why the phosphorylation leads to more disordered states of the switch domains than the WT KRAS. GTP produces HBIs with residues N116, D119, S145, A146, and K147 (Table 1 and Figure S10) but the phosphorylation hardly affects the stability of these hydrogen bonds.

**Figure 12:**
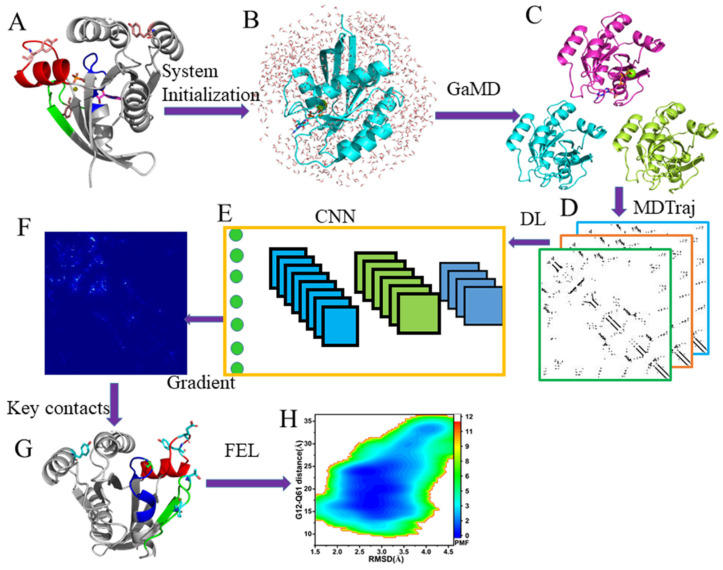
Workflow of deep leaning from GaMD simulations: (**A**) the GTP-bound KRAS with three phosphorylation sites, (**B**) the initialized GTP-bound KRAS, (**C**) conformational ensembles recorded in three independent GaMD trajectories, (**D**) images extracted though the MDTraj program, (**E**) convolution neural networks, (**F**) the saliency maps calculated through backward propagation, (**G**) key residue contacts identified by deep learning, and (**H**) free energy landscapes used for revealing the phosphorylation-mediated effect on free energy profiles of the GTP-bound KRAS.

Magnesium ion plays an important role in the GTP-KRAS binding and the function of KRAS. To reveal the effect of the phosphorylation on the stability of Mg^2+^, the distances involved in the Mg^2+^-mediated electrostatic interactions with residues and GTP were calculated (Figure 11 and Figure S11). The distances of Mg^2+^ away from the oxygen atom O of S17 fluctuate from 1.83 to 3.16 Å in the GTP-bound WT and pY137 KRAS (Figure 11A and Figure S11A) and they are located at 2.12 Å (Figure 11B); thus, pY137 scarcely influences the interaction of Mg^2+^ with the OG of S17. The distances of Mg^2+^ away from the OG of S17 transform between two different states in the pY32 and pY64 KRAS (Figure S11A) and their probabilities have two position sites (Figure 11B), suggesting that pY32 and pY64 slightly weaken the interaction between Mg^2+^ and the OG of S17. The distances of Mg^2+^ away from the oxygen atom O of T35 fall into a fluctuation range of 3.33–21.39 Å (Figure S11B), showing that the position of the SW1 relative to Mg^2+^ is greatly changeable. On the whole, pY137 enhances the electrostatic interaction of Mg^2+^ with the O of T35 (Figure 11C). The distances between Mg^2+^ and the mass center of OD1 and OD2 in D57 fluctuate from 1.48 to 3.49 Å in the GTP-bound WT, pY32, and pY64 KRAS while this distance falls into a range from 3.49 to 8.29 Å in the GTP-bound pY137 KRAS (Figure S11C). The distances of Mg^2+^ away from the mass center of OD1 and OD2 of D57 are distributed near 2.88 Å in the GTP-bound WT, pY32, and pY64 KRAS but it is populated at 4.01 and 4.91 Å in the GTP-bound pY137 KRAS (Figure 11D), suggesting that pY137 weakens the electrostatic interaction of Mg^2+^ with the carbonyl of D57. Based on Figure S11D and Figure 12E, the phosphorylation hardly produces the effect on the electrostatic interaction of Mg^2+^ with the oxygen atom O2B of GTP. The distances of Mg^2+^ away from the oxygen atom O2G of GTP transform between two different states in the GTP-bound pY32 and pY64 KRAS (Figure S11E). This distance is distributed at 1.86 Å in the GTP-bound WT and pY137 KRAS (Figure 11F). Although the first distance between Mg^2+^ and the O2G of GTP in the GTP-bound pY32 and pY64 KRAS is also located at 1.86 Å, its second value is distributed at 3.97 and 4.19 Å, separately (Figure 11F). As a result, pY32 and pY64 weaken the electrostatic interaction of Mg^2+^ with the O2G of GTP.

Through the above analyses, the phosphorylation-mediated influences on interaction networks are summarized: (1) the phosphorylation changes the salt bridge interaction between the nitrogen atom NZ and the phosphorus atom PB of GTP, (2) the phosphorylation reduces the occupancy of HBIs between GTP and the SW1, and (3) the phosphorylation produces a significant effect on the electrostatic interactions of Mg^2+^ with T35 and D57 located at the SW1 and SW2. The Mg^2+^-free state not only plays an important role in the nucleotide exchange of oncogenic KRAS mutants but also results in characteristic differences in the switch regions of the WT and mutated KRAS [79,80]. In the work of Hu et al., the 1D- and 2D-potential mean forces (PMFs) constructed by using the Mg^2+^-T35 distance as the RCs indicate that Mg^2+^ participates in the conformation transformation of RAS proteins [81]. Thus, the phosphorylation-mediated impacts on the HBIs of GTP with the SW1 and Mg^2+^ with T35 and D57 in the switch regions can regulate the activity of KRAS.

## 3. Materials and Methods

### 3.1. Scheme of Operating Calculations

MI-GaMD simulations, DL, and PCA were integrated to recognize key residue contacts and reveal phosphorylation-mediated effects on conformations of the GTP-bound KRAS and the overall scheme is shown in Figure 12. The operating procedure is as follows: (1) the initial atomics coordinates of systems were extracted from the protein data bank (PDB) and simulation systems were constructed using the Amber program to produce force field parameters, (2) three independent GaMD simulations were performed to relax conformations and collect conformational ensembles, (3) the MDTraj program was adopted to convert conformational ensembles into images for DL, (4) the images were randomly divided in the training set and validation set to perform image classification based on the two-dimensional (2D) convolutional neural network (CNN), (5) key residue contacts were identified through classic gradient-based pixel attribution [82], and (6) the reaction coordinates (RCs) were obtained using key residue contacts to construct FELs and reveal the energy basis for the phosphorylation-mediated effects.

### 3.2. Constructions of Simulated Systems

The complete structure of the GTP-bound WT KRAS is unavailable in the protein data bank (PDB). The GTP-bound WT KRAS was obtained by deleting GDP and incomplete KRAS from superimposed structures of the GDP-KRAS (PDB entry: 5W22) [83] and the GTP-KRAS (PDB entry: 5VQ2) [83]. To keep atomic coordinate consistence, residues Y32, Y64, and Y137 in the above obtained GTP-WT bound KRAS were, respectively, phosphorylated as pY32, pY64, and pY137 by using the Leap module in Amber22 to produce the GTP-bound pY32, pY64, and pY137 KRAS [84,85]. The magnesium ion (Mg^2+^) in the crystal structure was kept at the initial model. The protonated states of residues in KRAS were examined by using the program H++ 3.0 [86] and the rational protonation was assigned to each residue. All force field parameters used in our current study were assigned through the Leap module in Amber22 according to the procedure as follows: (1) the missing hydrogen atoms in the crystal were lined to their corresponding heavy atoms, (2) the parameters of KRAS were produced with the ff19SB force field [87] and the parameters of three phosphorylated resides were obtained from the work of Homeyer et al. [88], (3) the parameters of GTP was taken from the work of Meagher et al. [89], (4) an octahedral periodic box of water with a buffer of 12.0 Å was adopted to solve each of the GTP-bound WT, pY32, pY64, and pY137 KRAS, in which the force field parameters of water molecules were obtained from the TIP3P model [90] and (5) each simulated system was neutralized by adding sodium ion (Na^+^) in 0.15 M NaCl salt environment, in which in which the parameters of Mg^2+^, Na^+^, and Cl^−^ ions were derived from the study of Joung and Cheatham [91,92]. The corresponding information of the constructed systems was provided in Table S1.

### 3.3. Multiple Independent Gaussian Accelerated Molecular Dynamics

With the expectation for relieving bad contacts between atoms caused by initialization of four current GTP-KRAS systems, each system endured two-step minimizations consisting of 5000-cycle steepest descent minimization and 10,000-cycle conjugate gradient minimization. The optimized systems were slowly heated from 0 to 310 K within 2 ns in the canonical ensemble (NVT) with a weak harmonic restriction of 2 kcal mol^−1^‧Å2 on heavy atoms from the GTP-bound WT and phosphorylated KRAS. Then, four systems were further equilibrated at 310 K under the isothermal–isobaric ensemble (NPT). Subsequently, the 10-ns NPT conduction was executed to keep the density of the system in 1.01 g/cm^3^. Finally, independent 300-ns cMD production was implemented at the NVT with periodic boundary conditions and the particle mesh Ewald method (PME) [93], during which the initial atomic velocities of each structure were assigned by means of the Maxwell distribution. Four well-equilibrated systems were used as starting points for four independent GaMD simulations.

GaMD simulations adopt a harmonic boost potential to reduce free energy barriers in the biomolecules and enhance conformational samplings of systems. In GaMD simulations, if the potential energy $V\left(\overset{⃑}{r}\right)$ of the system is lower than a threshold energy *E*, the $V\left(\overset{⃑}{r}\right)$ is revised as $V^{*}(\overset{⃑}{r})$ according to the following Equations (1) and (2)

(1) $$V^{*}\left(\overset{⃑}{r}\right)=V\left(\overset{⃑}{r}\right)+∆V(\overset{⃑}{r})$$

(2) $$∆V\left(\overset{⃑}{r}\right)=\left\{\begin{array}{c} 0,\;\;\;\;\;\;\;\;\;\;\;\;\;\;\;\;\;\;\;\;\;\;\;\;\;\;\;\;V(\overset{⃑}{r})\geq E\\ \frac{1}{2}k{\left(E-V\left(\overset{⃑}{r}\right)\right)}^{2},\;\;\;V\left(\overset{⃑}{r}\right)<E \end{array}\right.$$

in the above equations, the parameter k represents the harmonic force constant, while the parameters *E* and *k* can be regulated by following the enhanced sampling principles defined in Equations (3) and (4) as below

(3) $$V_{max}\leq E\leq V_{min}+\frac{1}{k}$$

(4) $$k=k_{0}\frac{1}{V_{max-}V_{min}}$$

where if E is set as the lower bound $E=V_{max}$, then $k_{0}$ is determined with the Equation (5)

(5) $$k_{0}=\min(1.0,\;\;\frac{\sigma_{0}}{\sigma_{V}}\cdot \frac{V_{max}-V_{min}}{V_{max}-V_{avg}})$$

on the contrary, if E is set as the upper bound $E=V_{min}+\frac{1}{k}$, then $k_{0}$ is derived from the Equation (6)

(6) $$k_{0}=(1.0-\frac{\sigma_{0}}{\sigma_{V}})\cdot (\frac{V_{max}-V_{min}}{V_{avg}-V_{min}})$$

of which three energy parameters $V_{max}$, $V_{min}$, and $V_{avg}$ indicate the maximum, minimum, and averaged potential energies of the simulated systems extracted from the previous cMD simulations, separately. The parameter *σ_V_* corresponds to the standard deviation of the system potential energies and the *σ*_0_ is a user-determined upper limit for accurately reweighting. In our current study, 4-µs GaMD simulations, composed of four independent GaMD simulations of 1 µs, were run on the GTP-bound WT, pY32, pY64, and pY137 KRAS. To facilitate the DL and post-processing analysis, four independent GaMD trajectories were connected into a single GaMD trajectory (SGT) and the CPPTRAJ module inlayed in Amber 22 was applied to extract data for insight into the function of KRAS [94]. A program, PyReweighting, contributed by Miao et al. was employed to accurately reweigh and recognize the original free energy profiles of our current KRAS systems [95]. All cMD and GaMD simulations use the SHAKE algorithm to constrain chemical bonds connecting hydrogen atoms to heavy ones [96]. The Langevin thermostat with a collision frequency of 2.0 ps^−1^ was utilized to regulate the temperatures of four KRAS-related systems [97]. The PME method with a 12-Å cutoff was utilized to estimate the non-bonded interactions. The program pmemd.cuda implemented in Amber 20 [98,99] was employed to run all simulations. PCA was performed by using the CPPTRAJ module [94] to uncover the phosphorylation-mediated effect on dynamics behavior of KRAS and the details have been provided in our previous works [27,100].

### 3.4. Deep Learning

DL was utilized to decode the phosphorylation-mediated impacts on conformational dynamics of KRAS and our entire scheme, including system preparation, GaMD simulations, image extraction, DL, and construction of FELs, was depicted in Figure 2. The residue contact map in each GaMD trajectory snapshot was calculated by utilizing the Python packages MDTraj1.9.8.dev0 [101] and contact map explorer [101], in which a contact definition of ≤4.5 Å between any Cα atoms was adopted. Then, the obtained 170 × 170 residue contacts were converted into the 170 × 170-pixel grayscale images that can be identified by a two-dimensional (2D) convolutional neural network (CNN). Then, 80% and 20% of images randomly selected from 160,000 images extracted from the SGT by the MDTraj were, respectively, used for training and validation. The current 2D-CNN was constructed by using the Keras3.0 module [102] and the Python PyTorch package (https://pytorch.org/). The current CNN model was mainly composed of four convolutional layers of 3 × 3 kernel size, with 32, 32, 64, and 64 filters, individually, followed by three fully connected layers, the first two of which included 512 and 128 filters with a dropout rate of 0.5 each. The fully connected layer was the classification layer for discriminating four different KRAS. Apart from the classification layers, the“ReLu” activation function was utilized in all layers of the 2D-CNN, from which the“softmax” activation function was employed. A maximum pooling layer with 2 × 2 kernel size was followed after each convolutional layer. Lastly, the backpropagation by vanilla gradient-based pixel attribution [82] was applied to compute the saliency map of residue contact gradients so as to identify the function difference of KRAS caused by phosphorylation, in which residue contact was mapped onto the most populated structural cluster of each KRAS-related systems. We declare that our program was rewritten with the PyTorch package based on the work of Miao’s group [67] and a previous work has been performed [103].

### 3.5. Construction of Free Energy Landscapes

Key residue contacts were detected by the DL according to three following criterions: (1) the contact gradients were higher than 0.75 in the saliency maps, (2) the residue contacts were calculated by using all Cα atoms, and (3) obvious changes in contacts between structure domains can be captured. The distances between the Cα atoms of key residues recognized by the DL and root-mean-square deviations (RMSDs) of backbone atoms from KRAS were used as reaction coordinates (RCs) to reweigh GaMD simulations and construct FELs. 

In reweighting of GaMD simulations, the reweighted free energy $F\left(A\right)=-k_{B}Tln(\rho_{A})$ is computed as

(7) $$F\left(A\right)=F^{*}\left(A\right)-\sum_{k=1}^{2}\frac{\beta^{k}}{k!}C_{k}+F_{C}$$

where $F^{*}\left(A\right)=-k_{B}Tlnp^{*}\left(A\right)$ represents the modified free energy arising from GaMD simulations, $F_{C}$ indicates a constant, and ${\beta =k}_{B}T.$ The probability distribution $p^{*}\left(A\right)$ of selected RCs from GaMD simulations can be reweighted to recover the canonical ensemble distribution $\rho_{A}$. All calculations in the free energy reweighting were realized by using the program PyReweighting developed by Miao et al. and the detail for the reweighting procedure has been clarified in the work of Miao et al. [95]. 

## 4. Conclusions

The phosphorylation of different sites produces significant impacts on the activity of KRAS. Four independent GaMD simulations, each for running 1 μs, were conducted to probe the influences of the phosphorylation of pY32, pY64, and pY137 on conformational dynamics of the GTP-bound KRAS. The CNN-based DL was performed to identify significant functional domains and the results reveal that the switch domains are involved in obvious difference in structural contacts. The RCs learned by the DL were used to construct FELs and the results indicate that the phosphorylation not only induces more energy states than the WT KRAS but also leads to more disordered states of the switch domains. The information from the PCA suggests that the phosphorylation alters structural fluctuation in the switch domains. The interaction networks uncover that the phosphorylation changes not only the HBIs of the GTP with the SW1 but also the electrostatic interactions of Mg^2+^ with SW1 and SW2. The SW1 and SW2 participate in binding of KRAS to its effectors or regulators; hence, it is known that the alterations in conformational dynamics of the switch domains certainly affect the function of KRAS. This work is also anticipated to provide useful theoretical aids for deeply understanding the function of KRAS.

## Figures and Tables

**Table 1:** Hydrogen bonding interactions of GTP with KRAS identified by the CPPTRAJ program.

^a^ Hydrogen Bonds		^b^ Occupancy(%)			
Residues	GTP	WT	pY32	pY64	pY137
G13-N-H	O3B	89.2	88.1	89.3	87.2
V14-N-H	O1B	20.1	20.3	19.6	13.1
G15-N-H	O1B	99.0	98.3	97.6	99.5
K16-N-H	O1B	99.9	99.9	98.8	99.9
S17-N-H	O2B	99.5	96.2	87.3	88.8
A18-N-H	O1A	98.6	96.4	99.2	99.8
V29-O	O2′-HO’2	30.6	16.4	15.7	17.4
D30-O	O2′-HO’2	27.2	18.2	18.4	11.1
N116-ND2-HD21	N7	90.0	87.2	88.7	91.1
D119-OD1	N1-H1	91.1	93.3	93.1	90.4
D119-OD2	N1-H1	76.3	78.3	75.7	77.1
S145-OG-HG	O6	59.4	61.3	59.6	57.6
A146-N-H	O6	61.5	63.1	67.5	63.4
K147-N-H	O6	84.4	86.5	84.9	82.3

^a^ Hydrogen bonds are analyzed by an acceptor···donor distance of <3.5 Å and acceptor···H-donor angle of >120°. ^b^ Occupancy (%) is defined as the percentage of simulation time that a specific hydrogen bond exists.

## Data Availability

Data are contained within the article and Supplementary Materials.

## Supplementary Materials

The following supporting information can be downloaded at https://www.mdpi.com/article/10.3390/molecules29102317/s1. Table S1: The corresponding information for construction of simulation systems. Figure S1: Learning curves of the training and validation datasets for four GTP-bound KRAS systems; Figure S2: RMSDs of backbone atoms from the GTP-bound WT, pY32, pY64 and pY137 KRAS; Figure S3: Free energy profiles and representative structures of the GTP-WT KRAS; Figure S4: Geometric positions of key residues in representative structures of the GTP-pY32 KRAS; Figure S5 Geometric positions of key residues in representative structures of the GTP-pY64 KRAS; Figure S6: Geometric positions of key residues in representative structures of the GTP-pY137 KRAS; Figure S7: The time course of RMSDs for heavy atoms of GTP; Figure S8: The distances between the mass centers of all Cα atoms in the helix α2 and α3 and the distances between the mass centers of all Cα atoms in the helix α3 and α4; Figure S9: The function of eigenvalues as eigenvector indexes; Figure S10 Geometric position of key residues relative to GTP; Figure S11 The time course of distances for Mg^2+^-mediated electrostatic interactions: (A) the distances between Mg^2+^ and the oxygen atom OG of S17, (B) the distances of Mg^2+^ away from the oxygen atom O of T35, (C) the distances of Mg^2+^ away from the mass center of oxygen atoms of OD1 and OD2 in D57, (D) the distances of Mg^2+^ away from oxygen atom O2B of GTP and (E) the distances between Mg^2+^ and oxygen atom OG of GTP.
