# Supplementary material for: Molecular Mechanism of Phosphorylation-Mediated Impacts on the Conformation Dynamics of GTP-Bound KRAS Probed by GaMD Trajectory-Based Deep Learning
Source: Molecules. 2024 May 15;29(10):2317. doi: 10.3390/molecules29102317 (PMC11123822; doi:10.3390/molecules29102317)
Supplement: Supplementary file 1 [file molecules-29-02317-s001.zip › molecules-3002905-supplementary.pdf]

Molecular Mechanism of Phosphorylation-Mediated Impacts on the Conformation Dynamics of GTP-Bound KRAS Probed by GaMD Trajectory-Based Deep Learning

Jianzhong Chen [1,2,*], JianWang [1], Wanchun Yang [1], Lu Zhao [1], Juan Zhao [1] and Guodong Hu [2,*]

1 School of Science, Shandong Jiaotong University, Jinan 250357, China; wangjian_lxy@sdjtu.edu.cn (J.W.); yangwch1982@126.com (W.Y.); zhaolusdu@163.com (L.Z.); zhjuan2002@126.com (J.Z.)

2 Shandong Key Laboratory of Biophysics, Institute of Biophysics, Dezhou University, Dezhou 253023, China

* Correspondence: jzchen@sdjtu.edu.cn or chenjianzhong1970@163.com (J.C.); xzszhgd@163.com (G.H.)

Table S1. The corresponding information for construction of simulation systems

Systems	WT	pY32	pY64	pY137
Number of atoms	19735	19812	19713	19725
Number of Na^+	29	31	31	31
Number of Cl^-	21	21	21	21
Number of water molecules	5646	5670	5637	5641
Total charges	-8	-10	-10	-10
Simulation time	4-μs	4-μs	4-μs	4-μs
Number of frames	2000000	2000000	2000000	2000000

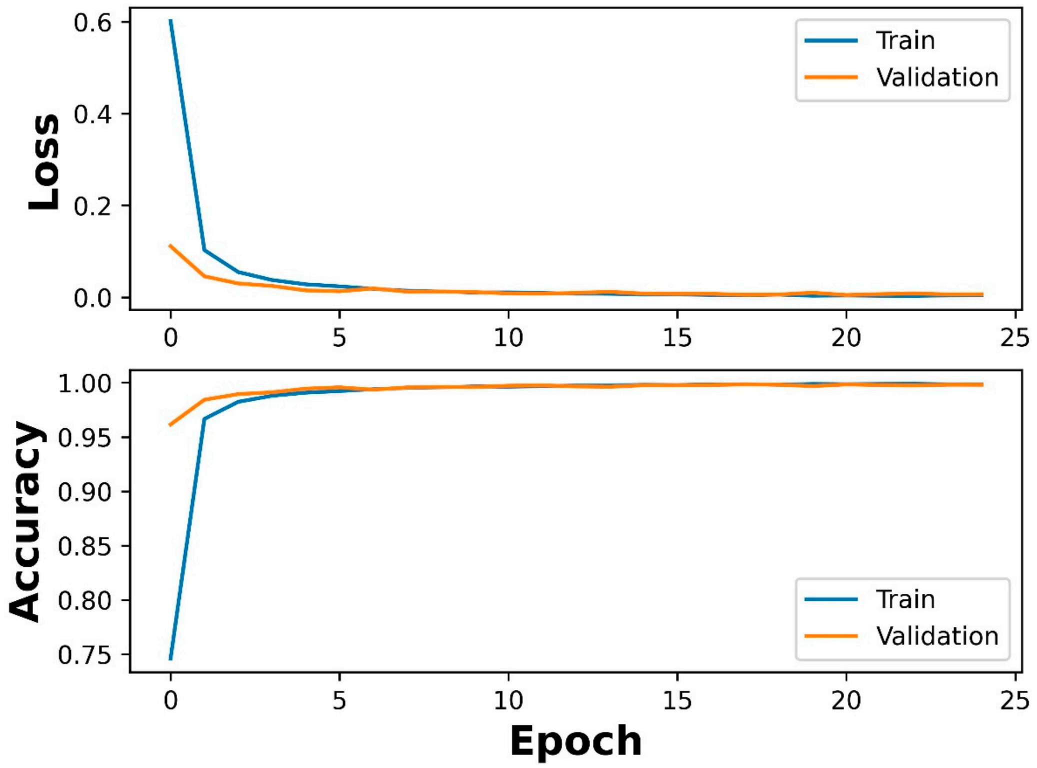

Figure S1. Learning curves of the training and validation datasets for four GTP-bound KRAS systems: (A) the loss and (B) the accuracy.

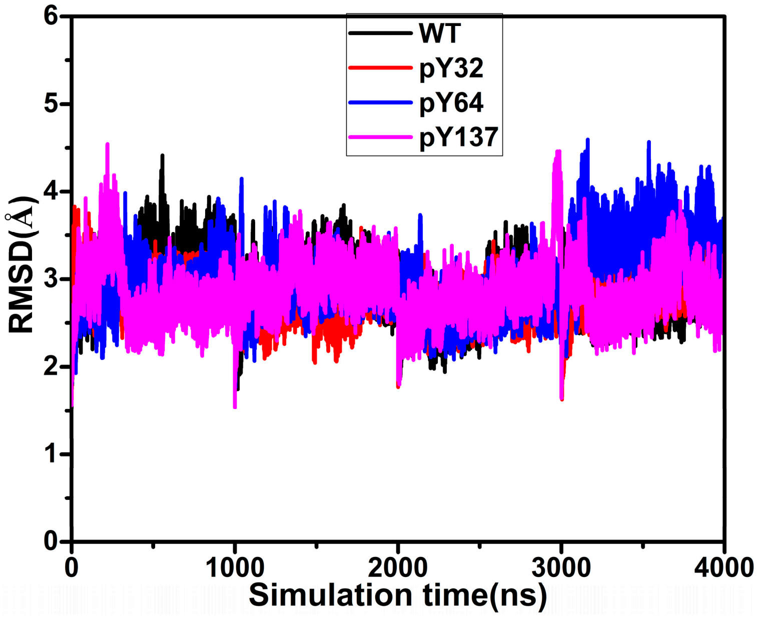

Figure S2. RMSDs of backbone atoms from the GTP-bound WT, pY32, pY64 and pY137 KRAS.

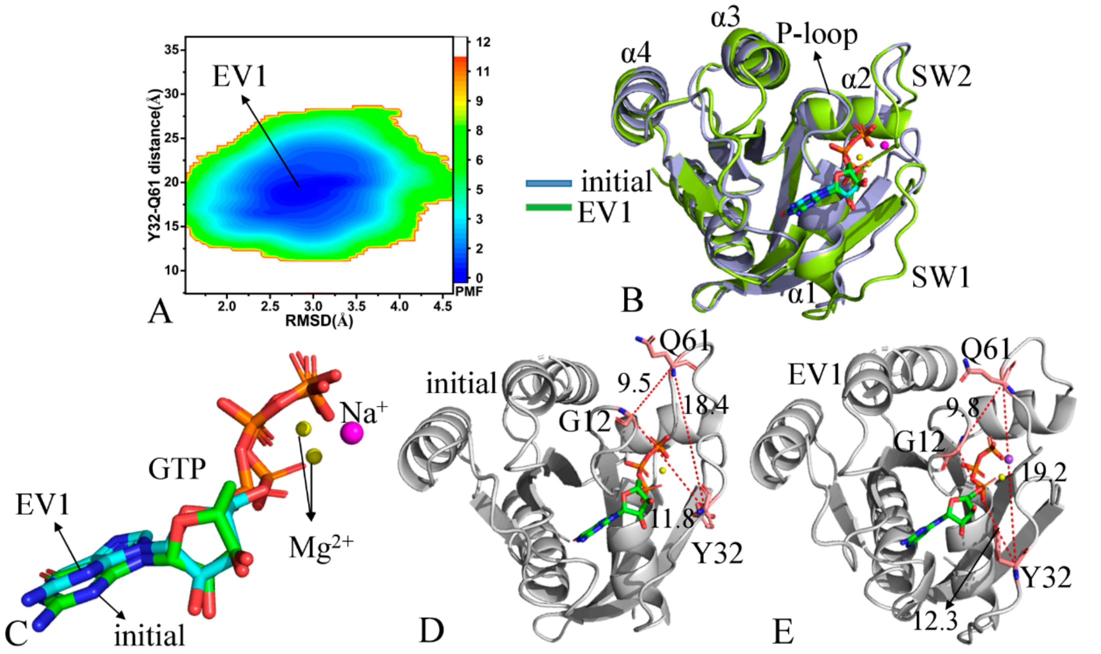

Figure S3. Free energy profiles and representative structures of the GTP-WT KRAS: (A) FEL, (B) superimposition of initial optimized structure with the EV1 structure, (C) structural superimposition of GTP and magnesium ions (Mg^{2+}) in the initially optimized structure and the EV1 structure, (D) geometric positions of key residues in the initially optimized structures and (E) geometric positions of key residues in the EV1 structure. The PMF is scaled in kcal/mol and the distances are scaled in Å.

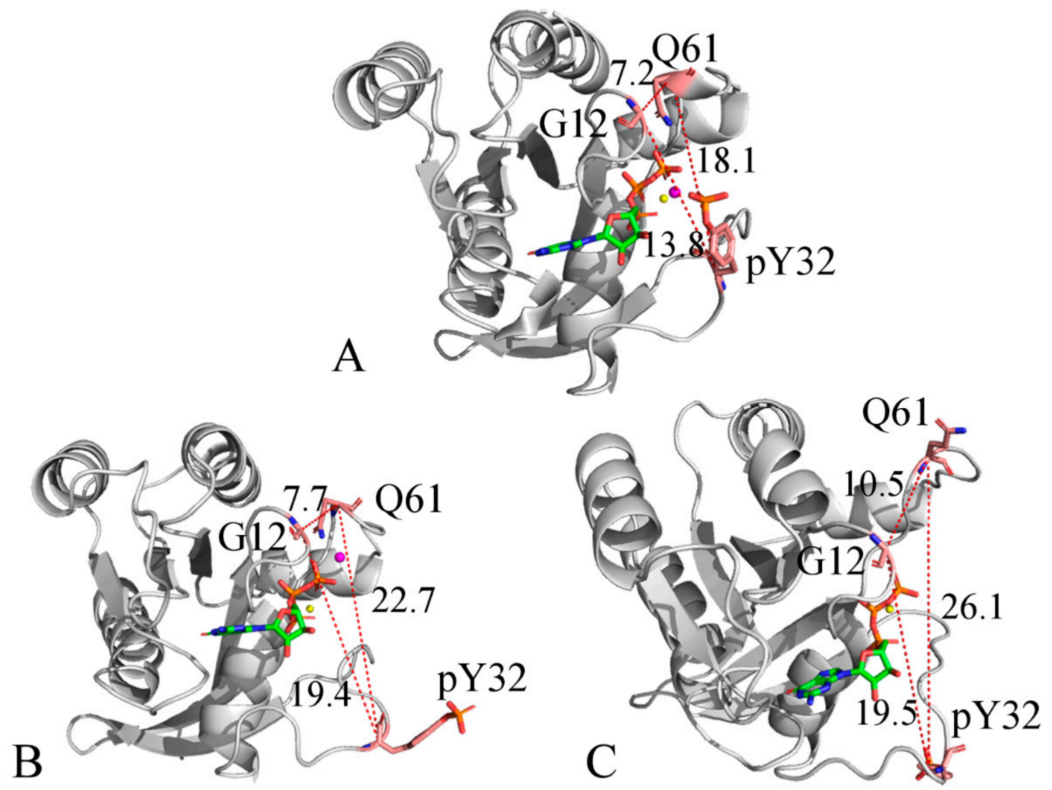

Figure S4. Geometric positions of key residues in representative structures of the GTP-pY32 KRAS: (A) the EV1 structure, (B) the EV2 structure and (C) the EV3 structure. The distances are scaled in Å.

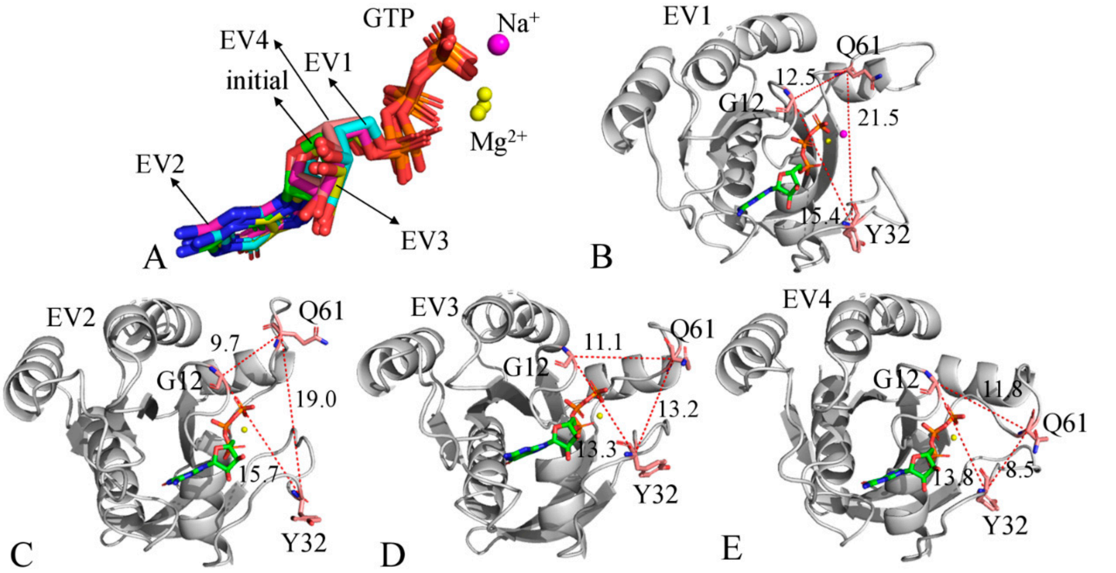

Figure S5. Geometric positions of key residues in representative structures of the GTP-pY64 KRAS: (A) structural alignments of GTP in the EV1-EV4 structures, (B) the EV1 structure, (C) the EV2 structure, (D) the EV3 structure and (E) the EV4 structure. The distances are scaled in Å.

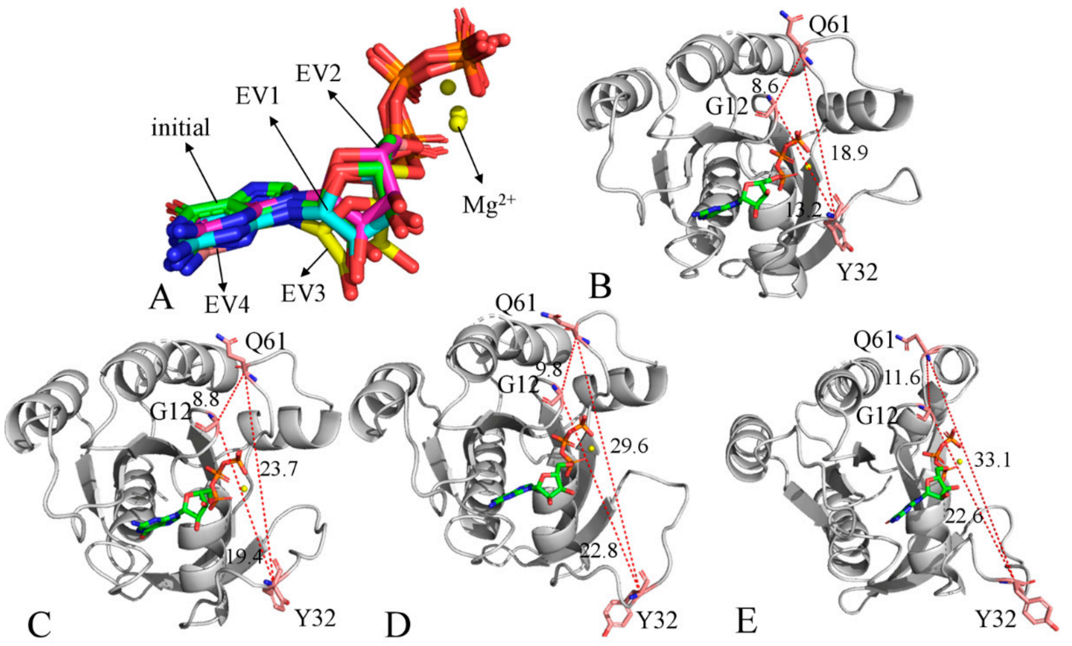

Figure S6. Geometric positions of key residues in representative structures of the GTP-pY137 KRAS: (A) structural alignments of GTP in the EV1-EV4 structures, (B) the EV1 structure, (C) the EV2 structure, (D) the EV3 structure and (E) the EV4 structure. The distances are scaled in Å.

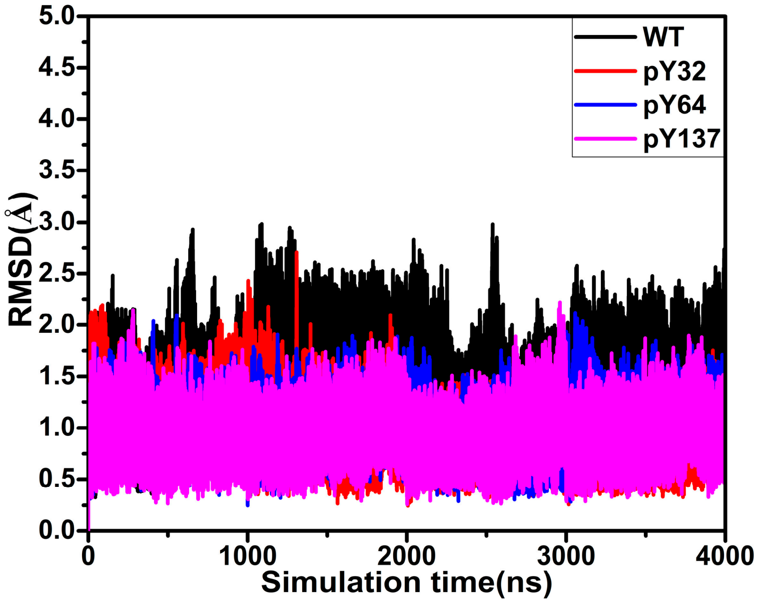

Figure S7. The time course of RMSDs for heavy atoms of GTP

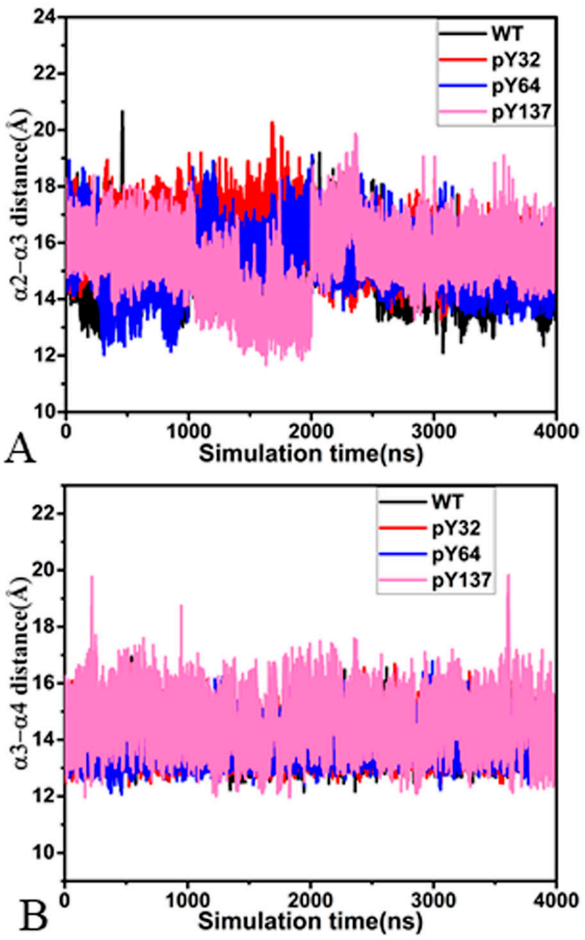

Figure S8 (A) The distances between the mass centers of all Cα atoms in the helix α2 and α3 and (B) the distances between the mass centers of all Cα atoms in the helix α3 and α4.

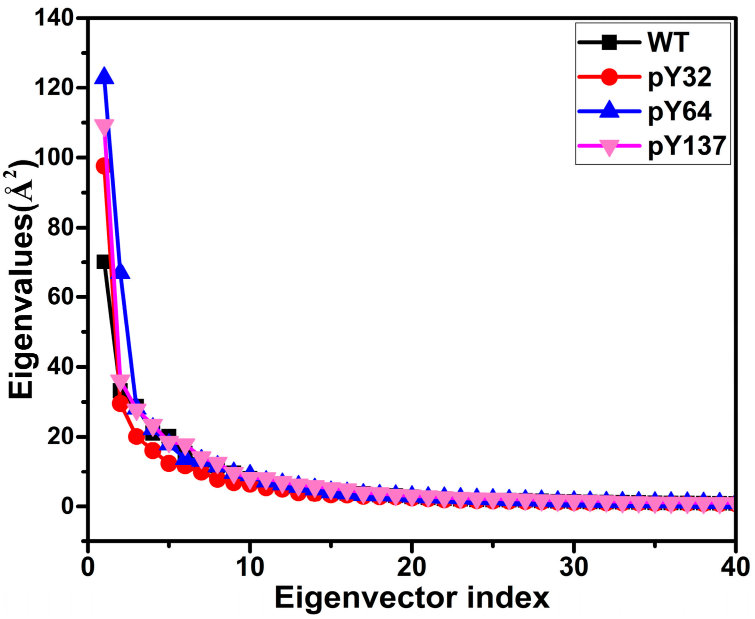

Figure S9. The function of eigenvalues as eigenvector indexes, which is used to describe the structural fluctuations along the eigenvector.

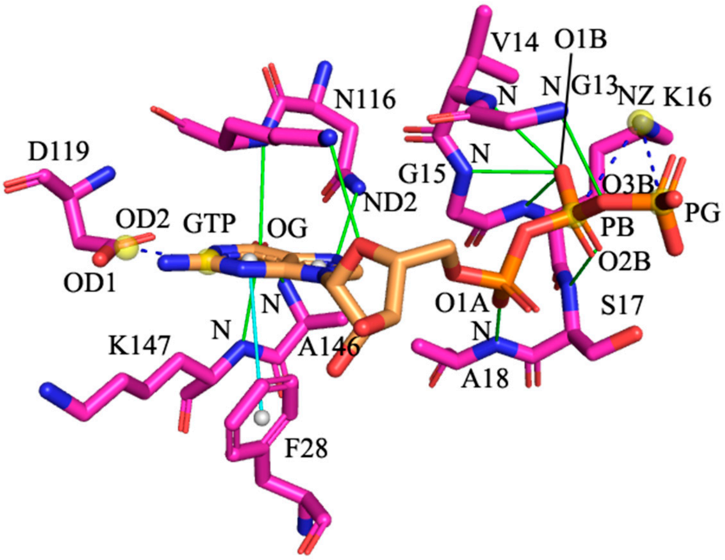

Figure S10. Geometric position of key residues relative to GTP.

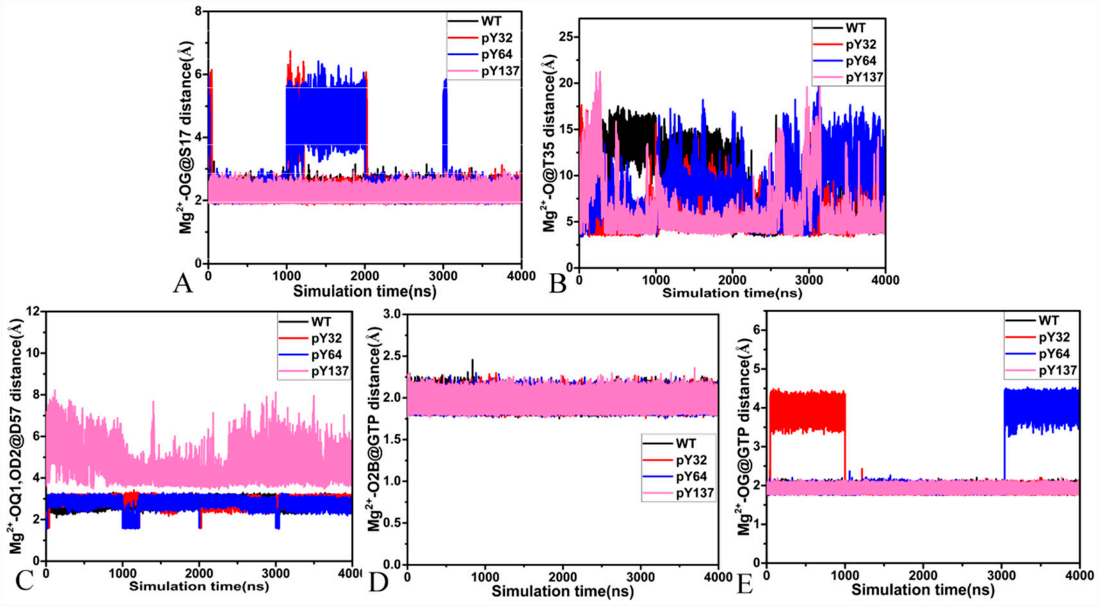

Figure S11. The time course of distances for Mg^{2+}-mediated electrostatic interactions: (A) the distances between Mg^{2+} and the oxygen atom OG of S17, (B) the distances of Mg^{2+} away from the oxygen atom O of T35, (C) the distances of Mg^{2+} away from the mass center of oxygen atoms of OD1 and OD2 in D57, (D) the distances of Mg^{2+} away from oxygen atom O2B of GTP and (E) the distances between Mg^{2+} and oxygen atom OG of GTP.
